# Adding Mandarin Peel Waste to a Biodegradable Polymeric Matrix: Reinforcement or Degradation Effect?

**DOI:** 10.3390/polym16223172

**Published:** 2024-11-14

**Authors:** Vincenzo Titone, Maria Chiara Mistretta, Luigi Botta

**Affiliations:** 1 INSTM Research Unit, Department of Engineering, University of Palermo, V. le delle Scienze, 90128 Palermo, Italy; vincenzo.titone@unipa.it; 2 Centro Interdipartimentale di Ricerca “Riutilizzo Bio-Based Degli Scarti da Matrici Agroalimentari” (RIVIVE), University of Palermo, V. le delle Scienze, 90128 Palermo, Italy

**Keywords:** biobased, biocomposite, biodegradable, circular economy, mechanical properties, rheology

## Abstract

In the current context, the use of fillers derived from fruit and vegetable waste is a crucial approach to mitigate waste and promote sustainable resource use, thus contributing to product life cycle completion and the achievement of sustainability goals. This study focuses on incorporating an endemic waste hitherto considered irrelevant within a biodegradable matrix. The resulting biocomposites were carefully characterized mechanically, rheologically, and morphologically to identify the connections between processability, structure, and properties. The results show that the presence of the filler results in an increase in the stiffness of the material (up to 27% in elastic modulus) accompanied by a decrease in tensile strength (approximately 50%) and elongation at break, which is on average about 7% at the highest filler content. This behavior was attributed to poor interfacial adhesion and the influence of a degradation process caused by the presence of citric acid and/or impurities in the filler.

## 1. Introduction

Over the years, polymers of fossil origin, such as polyethylene (PE), polypropylene (PP), polystyrene (PS), polyethylene terephthalate (PET), etc., have experienced rapid growth due to their use in various industries, such as packaging, building, automotive, etc. However, it is well known that these materials cause various problems related to environmental pollution [1,2,3]. Consequently, to minimize these problems, various biopolymers have been developed in recent years. Biopolymers, or bioplastics, according to European Bioplastic, ref. [4] are defined as such if they are biobased, biodegradable, or features both properties. Among the various types of biopolymers, those based on polylactic acid and starch are the most commercially available, followed by polyesters. However, these materials, compared to other industrial plastics, have much higher costs. Incorporating different fillers to reduce costs and improve properties [5,6] has always attracted researchers and industry, all the more so in an age when sustainable production and consumption have become a prerogative to avoid catastrophic scenarios. In this context, the agrifood industry emerges as a crucial sector, as it is responsible for generating a significant amount of by-products that can potentially be used as fillers. In fact, with this in mind, various food wastes such as mangoes [7], bananas [8], and others [9,10,11,12,13,14,15] are beginning to be incorporated into various biodegradable matrices to retain the advantage of biodegradability. In fact, as highlighted in several reviews [16,17,18,19,20], the use of these fillers is considered essential to promote the development of sustainable material solutions. However, little attention is paid to citrus waste today. Traditionally considered waste, citrus waste contains a number of bioactive compounds, such as polyphenols, flavonoids, and fibers, which can be exploited for a variety of purposes [21,22]. For example, mandarin peels contain a significant amount of plant fibers, which can be used to improve the mechanical strength of a material as well as to offer significant benefits in terms of sustainability, waste reduction, and efficient use of resources. This underscores the importance of carefully considering the potential of each food waste.

In this context, the aim of this paper is to evaluate the use of mandarin peels powders, obtained by grinding an endemic peel, as a filler in the formulation of biocomposites. This evaluation is also aimed at addressing the current issue of food waste management, which is a significant challenge for the agri-food sector. In this regard, biocomposites were prepared with concentrations of 10% and 20% by weight using a biodegradable blend known by the trade name Bio-Flex^®^ as the matrix. This blend, which consists of poly(lactic acid) (PLA) and poly(butylene adipate co-terephthalate) (PBAT), has attracted considerable interest in the packaging industry both as a biodegradable alternative to low-density polyethylene (LDPE) and for having recently been approved for food contact [23]. Furthermore, the choice of this blend is in line with EU rules on packaging design and waste management [24], which aim to promote more sustainable packaging solutions. In fact, as reported in our previous study [23], this blend offers good recyclability, making it a suitable option for this work and in line with EU sustainability goals.

Mandarin peel powders (MPPs), on the other hand, were obtained by grinding Tardivo di Ciaculli, a variety endemic to the Palermo area [25] (previously oven-dried at 90 °C for 48 h). Finally, morphological, mechanical, and rheological characterizations were performed on the biocomposites obtained in order to find correlations between processability, structure, and properties.

## 2. Materials and Methods

### 2.1. Material

The sample used in this work was a commercially available grade known as Bio-Flex^®^ F2110 (FKuR Kunststoff GmbH, Willich, Germany), which we abbreviate as BF, with the following main properties: melt flow index (MFI) = 6 g/10 min (at 190 °C and 2.16 kg), density = 1.27 g/cm^3^, and melting point = 153 °C.

Mandarin peel powders (MPPs) were obtained by granulating mandarin peels from the agri-food industry with an automatic mortar. In more detail, the species used was an endemic species of the Palermo area known as Tardivo di Ciaculli [25]. Before grinding, the peels were dried using a laboratory vacuum oven for 48 h at a temperature of 90 °C.

**Figure 1:**
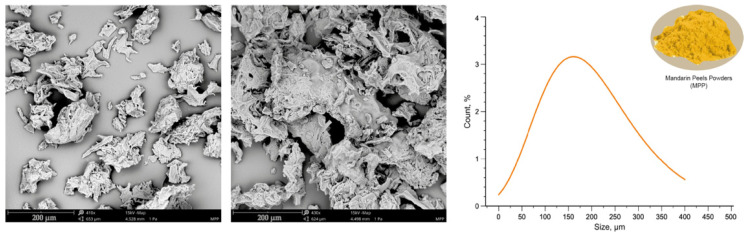
SEM images of mandarin peels powder particles and normal distribution curve.

Figure 1 shows that the particle size range was between 20 μm and 400 μm.

### 2.2. Preparation of BF/MPP Biocomposites

Biocomposites were prepared by adopting a corotating twin-screw extruder (OMC, Saronno, Italy) with a screw diameter of 19 mm and L/D ratio of 35 mm. BF and MPP were first mixed in the solid state and then fed into the extruder. The temperature profile used was 130-140-150-160-170-180-180 °C, the screw speed was set to 120 rpm, and the gravity feeder was set to 10 rpm. The melt at the extruder outlet was cooled in line in a water bath, pelletized, and then used for future characterizations. Before processing, the materials were dried at 60 °C for BF and 80 °C for MPP overnight to avoid hydrolysis [26].

**Figure 2:**
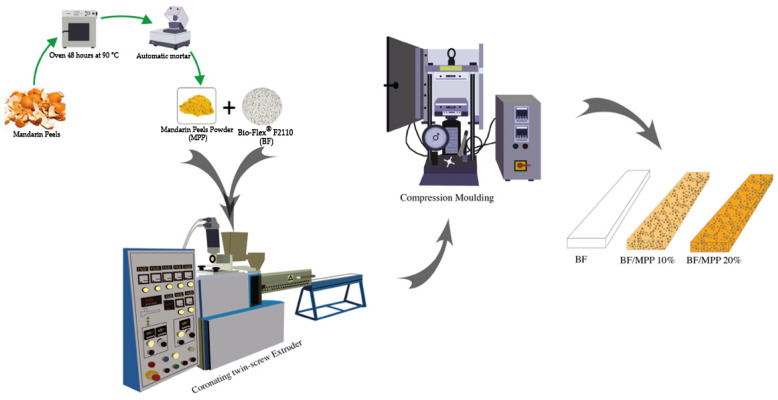
Scheme of the procedure used in this work.

Figure 2 shows an example of a scheme for obtaining the powders to making the biocomposites.

All samples for the subsequent analyses were obtained by compression molding with a Carver laboratory hydraulic press (Carver, Wabash, IN, USA) at a temperature of 180 °C with a mold pressure of 300 psi for about 3 min, as shown in Figure 2.

### 2.3. Characterization

#### 2.3.1. Dynamic Mechanical Thermal Analysis (DMTA)

Dynamic mechanical thermal analysis was performed using a Metravib DMA 50 (Metravib, Limonest, France). The measurements were carried out at a constant frequency of ω = 1 Hz at a temperature range of 35 to 90 °C with a heating rate of 3 °C/min. Analyses were carried out on samples cut to the size of 10 × 30 × ≈ 0.5 mm before being mounted in the DMTA apparatus. Before testing, all the samples were left to dry under vacuum for 4 h at 60 °C.

#### 2.3.2. Differential Scanning Calorimetry (DSC) Analysis

Thermal analysis was performed using differential scanning calorimetry (DSC) using a Chip-DSC 10 (Linseis Messgeraete GmbH, Selb, Germany). The amount of sample placed in the DSC aluminum pans was about 8 ± 3 mg, while the heating rate was from 10 °C/min up to 200 °C.

#### 2.3.3. Mechanical Characterization

The tensile properties were analyzed using an Instron Universal Testing Machine (Instron, High Wycombe, UK) mod. 3365 equipped with a 1 kN load cell. Tensile strength specimens were rectangular plates obtained by compression molding, as reported above, according to ASTM D638-14 [27].

#### 2.3.4. Morphological Analysis (SEM)

Morphological analysis of the samples was performed by Phenom proX scanning electron microscope (Phenom World, Eindhoven, The Netherlands). Samples for analysis were obtained by immersing the specimens in liquid nitrogen for about 30 min and breaking them up in a brittle manner. Before analysis, each sample was gold spattered to make them conductive.

#### 2.3.5. Rheological Characterization

Rheological tests were performed with an ARES G2 (TA Instruments, New Castle, DE, USA) equipped with a parallel plate geometry (diameter 25 mm). Measurements were carried out over an angular frequency range of 0.1 to 100 rad/s at a temperature of 180 °C.

#### 2.3.6. Intrinsic Viscosity

The intrinsic viscosity of PLA, PBAT, and their biocomposites was measured with an iVisc LMV 830 capillary viscometer (Lauda Proline PV 15, Lauda Konigshofen, Germany).

The biocomposites were separated from MPP by dissolving them in tetrahydrofuran (THF) for 12 h at room temperature under stirring in a magnetic stirrer and then filtered with the help of a water vacuum pump. Thereafter, the separated polymer was dried and then solubilized again in THF under stirring for 1 h in order to prepare a solution with a concentration equal to 0.2% (wt/wt).

Intrinsic viscosity values were then calculated using the Solomon–Ciuta equation [28]:

(1) $$\left[\eta \right]=\frac{\sqrt{2\;\left(\eta_{s}-\ln\eta_{r}\right)}}{C}$$

where C is the concentration of the polymer solution and η_s_ and η_r_ are the specific and relative viscosities, respectively. The viscosity of each sample solution was obtained from the average of three flow measurements.

The viscosimetric average molecular weight, M, was calculated with the Mark–Houwink equation:

(2) $$\left[\eta \right]=K\;M^{\alpha }$$

where K and α depend on the specific polymer–solvent system. In this case, PLA in THF and PBAT in THF at 30 °C are K = 0.0174 and 0.0150 mL/g, respectively, and α = 0.736 and 0.776, respectively [29,30].

#### 2.3.7. Statistical Analysis

Tensile test data were analyzed using one-way analysis of variance. Student’s *t*-test was used to compare date. The statistical significance level was set at *p* < 0.05

## 3. Results and Discussion

**Figure 3:**
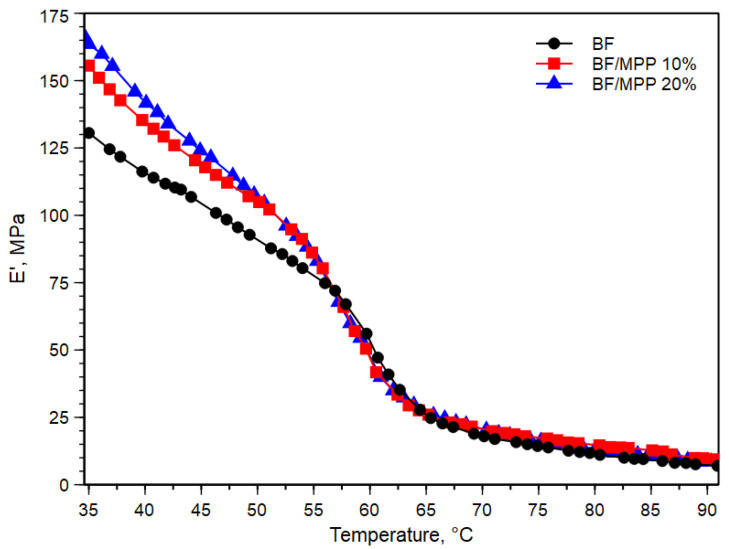
Storage modulus (E′) against temperature of BF and their biocomposites.

Figure 3 shows the plot of storage modulus, E′, against the temperature of BF and their biocomposites.

The value of E′ at a temperature of about 35 °C showed an increase proportional to the increase in filler content. Specifically, E′ increased from 131 MPa to 156 and 164 MPa for BF/MPP 10 and BF/20MPP, respectively. This can be attributed to the stiffness of MPP and, notably, the restricted mobility of BF chains due to the filler. As the temperature increased, all systems displayed a decrease in E′ around 60 °C, which is associated with the glass transition temperature (Tg). This reduction in modulus was slightly less pronounced with higher filler content, with the curves remaining above that of the matrix, clearly indicating the filler reinforcing effect. This impact of the filler on the modulus of the biocomposites, or its effectiveness, can be further represented by a coefficient “C”, which can be calculated using the following equation [31]:

(3) $$C=\frac{({E_{g}^{\prime }/E_{r}^{\prime })}_{composits}}{({E_{g}^{\prime }/E_{r}^{\prime })}_{resin}}$$

where $E_{g}^{\prime }$ and $E_{r}^{\prime }$ are the storage modulus values in the glass and rubbery region, respectively. Table 1 displays the coefficient C of the two BF/MPP biocomposites.

It can be observed that the C value decreased with increasing filler content. Generally, a higher C value indicates that the filler is less effective. However, although the values were very close to each other, this decreasing trend suggests that the effectiveness was better with higher MPP content, where the maximum stress transfer between the filler and the matrix occurred.

**Figure 4:**
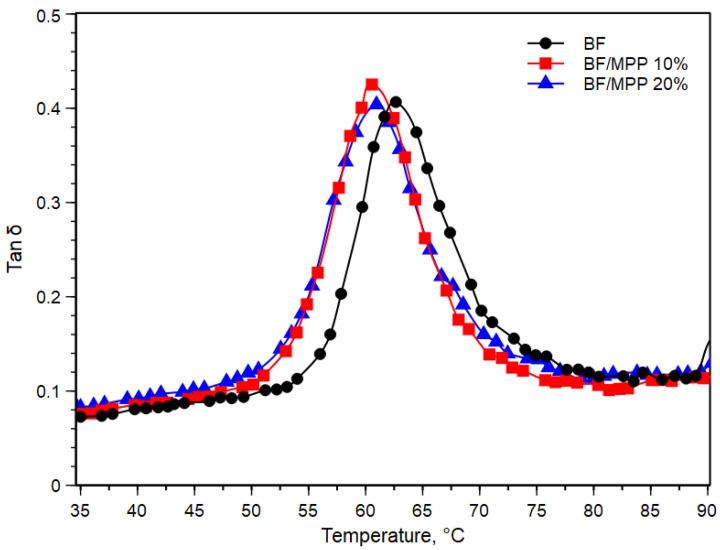
Damping factor (Tan δ) against temperature of BF and their biocomposites.

An important parameter to characterize the viscoelasticity and damping capacity is the tan δ, which indicates the ratio between the loss modulus and the storage modulus during a dynamic loading cycle. Figure 4 shows the behavior of the variable tan δ as a function of temperature.

**Figure 5:**
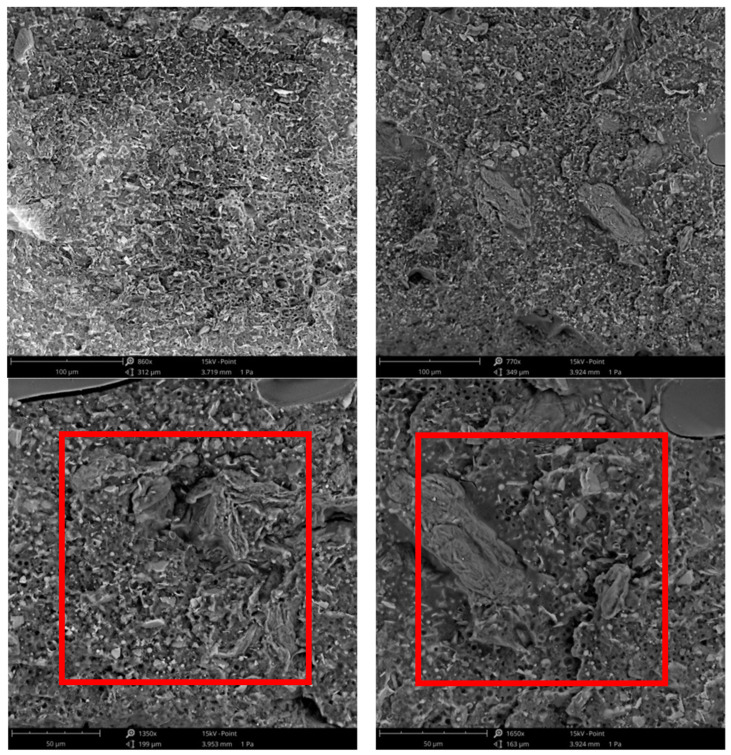

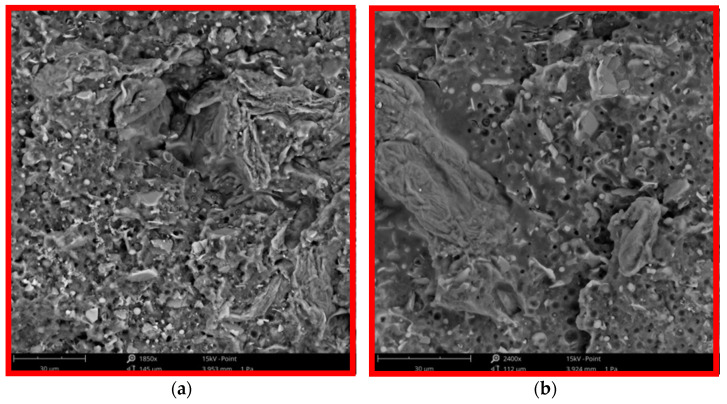
SEM micrographs at different magnifications: (**a**) BF/MPP 10%; (**b**) BF/MPP 20%.

As visible in Figure 4, a shift of the Tan δ peak toward lower temperatures was observed, consistent with what has been observed in similar systems [32,33]. This phenomenon implies that the mobility of BF was impaired due to the increased interfacial weakness resulting from the homogeneous dispersion capability of the filler. Indeed, this poor interfacial adhesion between the polymer and the filler was further confirmed by the SEM analysis shown in Figure 5, thus confirming the interpretation of the Tan δ peak shift.

In keeping with the results of the DMTA analysis, the data obtained from the thermal analysis, summarized in Table 2, confirmed the previous observation of a decrease in the glass transition temperature (Tg). However, it is interesting to note a slight increase in enthalpy values, from 7.1 ± 0.4 for BF to 9.8 ± 0.7 and 11.7 ± 1.2 for BF/MPP 10 and BF/20MPP, respectively. This change has been previously reported in other works [10,34,35] using biodegradable polymers as matrices and is usually attributed to the nucleating action of the filler on the polymer chains. An additional item of interest is the slight increase in melting temperature as the concentration of MPP increased. This result can be attributed both to a slight nucleation effect and to a general decrease in molecular weight that led to an increase in crystallinity. Indeed, this result is also in agreement with what has been observed in similar systems [10,34,35], reinforcing the idea that the presence of the filler significantly affects the thermal behavior of the composite.

**Figure 6:**
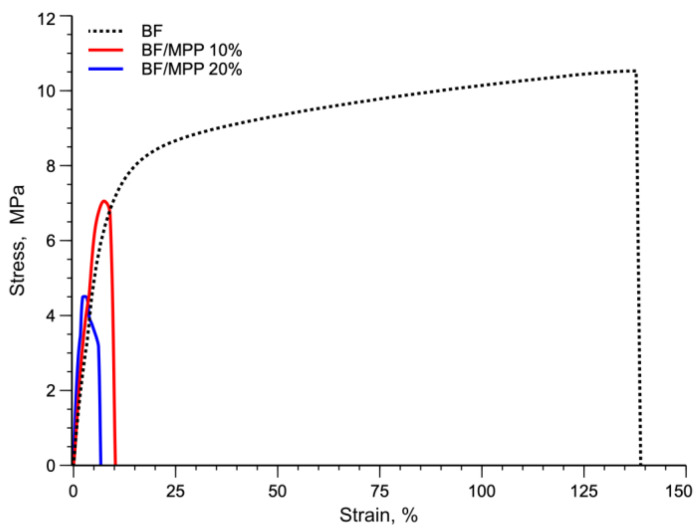
Typical stress–strain curve of BF and biocomposites.

Figure 6 shows the typical stress–strain curves of BF, BF/MPP 10, and BF/MPP 20.

The average values for the elastic modulus, tensile strength, and elongation at break for the BF sample were 129 ± 7.2 MPa, 10.2 ± 1.2 MPa, and 138 ± 21%, respectively. As can be seen from the stress–strain curves, the addition of MPP caused a slight increase in elastic modulus, in accordance with the results of DMTA tests, but a drastic decrease in both tensile strength and especially elongation at break. In detail, the elastic modulus increased by about 17% and 27% for BF/MPP 10% and BF/MPP 20%, respectively. Tensile strength and elongation at break decrease from 10.2 ± 1.2 MPa and 138 ± 21% for BF to 7.6 ± 0.8 MPa and 12 ± 3.4% for BF/MPP 10% and 4.7 ± 0.7 MPa and 6.6 ± 1.1% for BF/MPP 20%. These decreases in tensile strength and elongation at break can be attributed to premature failure of the specimens due to limited interfacial adhesion, as demonstrated by the SEM micrograph in Figure 5. In fact, consistent with previous works [10,36], it has been found that this premature failure during mechanical testing is a result of limited interfacial adhesion between the polymer and the matrix, which is responsible for stress concentrations and poor transfer of particle loads to the matrix. Table 3 summarizes the values of the elastic modulus E, tensile strength TS, and elongation at break EB of all the systems investigated, with their standard deviations.

**Figure 7:**
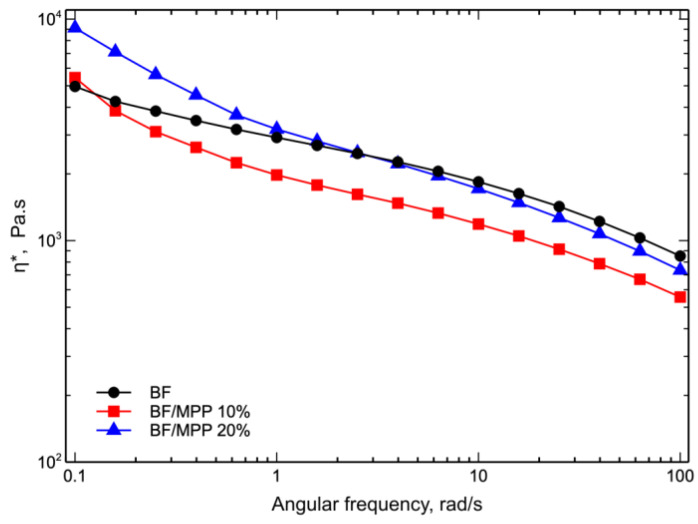
Complex viscosity (η*) as a function of frequency of BF and biocomposites.

Figure 7 shows the complex viscosity, η*, curves as a function of angular frequency of the BF and biocomposites.

**Figure 8:**
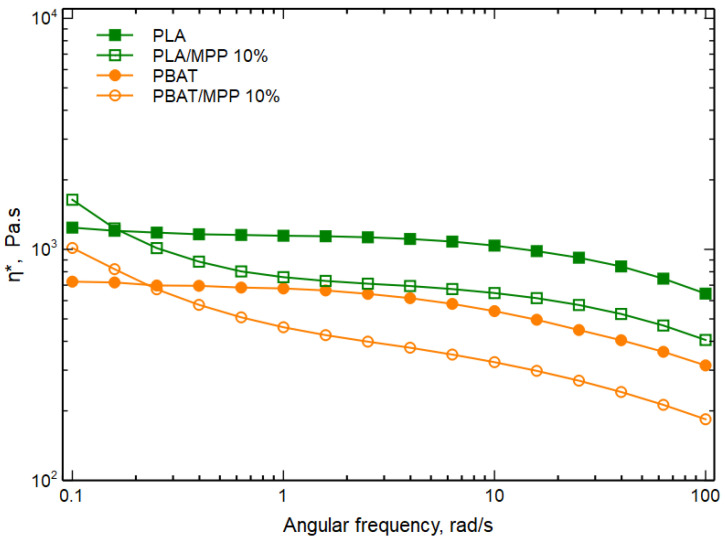
Complex viscosity (η*) as a function of frequency of PLA, PBAT, and their biocomposites.

As highlighted in Figure 7, the two biocomposites exhibited non-Newtonian behavior at low frequencies instead of the typical Newtonian plateau. This non-Newtonian behavior became more pronounced with increasing MPP content, exhibiting the typical characteristics of a pseudoplastic fluid. This peculiarity, commonly known as “shear thinning”, was expected and consistent with previous research on reinforced composites [10,37,38]. Unexpectedly, however, an unusual behavior emerged as the angular frequency increased. In fact, when the angular frequency exceeded 0.1 rad/s, the complex viscosity curve of the BF/MPP 10 sample showed lower viscosity than that of the matrix. Similarly, the BF/MPP 20 sample showed similar behavior, but with an angular frequency exceeding 1 rad/s. In this regard, the behavior of individual blend components was analyzed separately, as shown in Figure 8, with the flow curves of PLA, PBAT, and their biocomposites.

The curves of the two biocomposites (PLA/MPP 10% and PBAT/MPP 10%) show a similar trend from that observed previously in the blend. This consistency in the results was further investigated through the use of capillary viscosimetry. As described in the experimental section, the Solomon–Ciuta and Mark–Houwink equations were used to determine intrinsic viscosity (η) and molecular weight (Mw), respectively, with the results shown in Table 4.

From Table 4, a clear decrease in the intrinsic viscosity of PLA/MPP 10% and PBAT/MPP 10% compared with pure PLA and PBAT can be seen. Obviously, these results coincided with the decrease in molecular weight, since molecular weight usually shows a direct correlation with intrinsic viscosity. These observed reductions in molecular weight can be associated with the degradative effect of the matrix, which is attributable to the presence of citric acid [39] and/or impurities in the mandarin peels. In fact, as shown in similar studies [39,40,41], the presence of acids and/or impurities in these wastes could likely act as catalysts or accelerate the degradation process of these biodegradable polymers. Consequently, the lower elongation at break values found may be attributed not only to the limited interfacial adhesion between the polymer and the filler but also to the degradation effect caused by the presence of the filler. On the other hand, the increase in modulus observed in these biocomposites, related to the influence of MPP, increases the stiffness of the material, offering a viable solution for rigid packaging systems. This result could assume a significant role for sustainability as, in the face of the growing problem of packaging waste from non-renewable resources, its use contributes to a more sustainable product life cycle.

**Figure 9:**
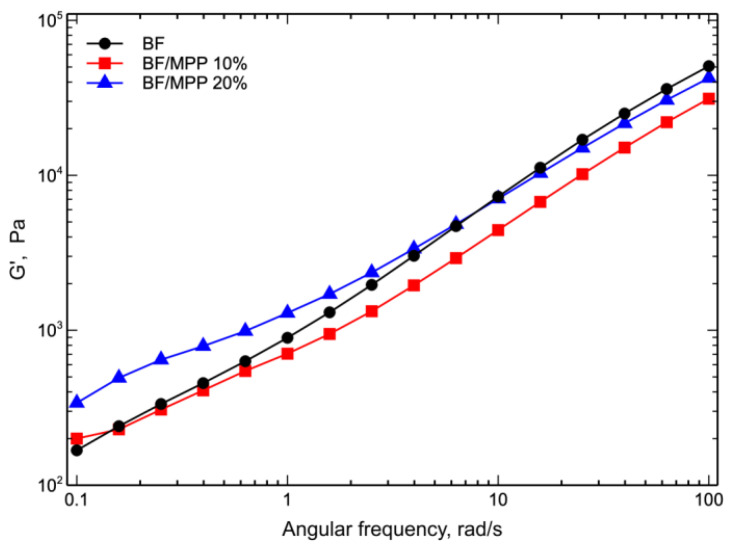
Storage modulus (G′) as a function of frequency of BF and biocomposites.

Figure 9 shows the storage modulus (G′) as a function of angular frequency of BF and biocomposites.

The curves of the storage modulus, G′, accurately reflect the complex viscosity trend, where the increase in viscosity caused by MPP particles at low frequencies results in an increase in the storage modulus. In particular, there was a gradual increase in the storage modulus as the MPP content increased, which became almost independent of frequency. This feature, previously observed in other composite systems refs. [38,42,43,44], suggests a transition in the viscoelastic behavior of the melt to a solid-like behavior.

## 4. Conclusions

In this paper, the incorporation of an endemic waste previously considered unimportant, such as mandarin peels, within a biodegradable polymer blend was studied. All biocomposites produced were subjected to rheological, morphological, and mechanical analyses. The results indicated that the E′ curves at room temperature increased with increasing MPP, with a smaller decrease with increasing temperature than those of the matrix, indicating higher thermal stability. However, premature failure of the samples was observed due to poor interfacial adhesion, which caused a drastic decrease in elongation at break at the highest filler content. Moreover, an increase in enthalpy values was observed due to nucleating action of the filler. In rheological terms, the particles induced an increase in viscosity at low frequencies, but this was followed by a drastic decrease with increasing angular frequency, bringing the values below the matrix. This effect, probably degrading, can be attributed to the presence of citric acid and/or impurities in the mandarin peel powder. In fact, citric acid and/or impurities, acting as catalysts, caused a marked decrease in intrinsic viscosity and molecular weight as observed in tests performed on the pure components of the blend. In summary, these results still raise open questions on the use of biomass as fillers for the development of biocomposites.

## Figures and Tables

**Table 1:** The Coefficient C of the two BF/MPP biocomposites.

Sample Code	C
BF/MPP 10%	0.958
BF/MPP 20%	0.918

**Table 2:** DSC results of glass temperature (Tg), melting temperature (Tm), and fusion enthalpy (∆H) for BF and biocomposites.

Sample Code	T_glass_, °C	T_melt_, °C	∆H, g/J
BF	63 ± 1.1	154 ± 1.2	7.1 ± 0.4
BF/MPP 10%	61 ± 0.8	155 ± 1.6	9.8 ± 0.7
BF/MPP 20%	59 ± 0.6	159 ± 1.8	11.7 ± 1.2

**Table 3:** Values of elastic modulus E, tensile strength TS and elongation at break EB with their standard deviations of BF and biocomposites.

Sample Code	E, MPa	T_S,_ MPa	EB, %
BF	129 ± 7.2 ^a^	10.2 ± 1.2 ^a^	138 ± 21 ^a^
BF/MPP 10%	151 ± 9.7 ^b^	7.6 ± 0.8 ^b^	12 ± 3.4 ^b^
BF/MPP 20%	164 ± 12 ^b^	4.7 ± 0.7 ^c^	6.6 ± 1.1 ^c^

Letters indicate significant differences (*p* < 0.05) when analyzed by multiple Student’s *t*-tests.

**Table 4:** Intrinsic viscosity (η) and molecular weight (Mw) values of PLA, PBAT, and their biocomposites.

Property	PLA	PLA/MPP 10%	PBAT	PBAT/MPP 10%
η, dL/g	1.14 ± 0.03	0.57 ± 0.01	0.64 ± 0.02	0.23 ± 0.01
Mw, Da	1.54 × 10^5^	5.97 × 10^4^	4.80 × 10^4^	1.26 × 10^4^

## Data Availability

The original contributions presented in the study are included in the article, further inquiries can be directed to the corresponding authors.
